# Platelet counts, but not neutrophil counts, are associated with non-bleeding-related mortality in patients with hematological malignancies and post-chemotherapy febrile neutropenia

**DOI:** 10.1016/j.htct.2026.106498

**Published:** 2026-07-10

**Authors:** Gabriela Romantini Salioni, Carla Roberta Peachazepi Moraes, Leticia Queiroz da Silva, Edwin Mayr, Bruno Kosa Lino Duarte, Erich V De Paula

**Affiliations:** a School of Medical Sciences, University of Campinas, Rua Vital Brasil, 80, Cidade Universitária, CEP: 13.083-888, Campinas, SP, Brazil; b Pontifical Catholic University of Campinas (PUC-Campinas), Rua Professor Doutor Euryclides de Jesus Zerbini, 1516, CEP: 13087-571 Pq. Rural Fazenda Santa Cândida, Campinas, SP, Brazil; c Hematology and Hemotherapy Center, University of Campinas, Rua Carlos Chagas, 480 - Barão Geraldo, Campinas, SP, Brazil

**Keywords:** Febrile neutropenia, Sepsis, Neutrophils, Platelets

## Abstract

**Introduction:**

Neutrophils and platelets are key elements of the host immune response to sepsis, participating in both pathogen clearance and in sepsis-related tissue damage. Although it is widely recognized that patients with hematological malignancies and chemotherapy-induced febrile neutropenia are at increased risk of severe sepsis complications, the prognostic impact of neutrophil and platelet counts on sepsis-related outcomes remains controversial, with conflicting results regarding the magnitude of neutropenia. Moreover, to our knowledge, no studies have addressed the association of thrombocytopenia with outcomes in febrile neutropenia in patients with hematological malignancies. This study investigated the association of neutropenia and thrombocytopenia in a consecutive cohort of 113 patients with underlying hematological malignancies who were admitted to an academic tertiary hospital with chemotherapy-induced neutropenia.

**Methods:**

Neutrophil and platelet counts were obtained at fever onset and 24, 48 and 72 hours thereafter.

**Results:**

Neutrophil counts at the time of febrile neutropenia were not associated with sepsis-related mortality or other negative outcomes. However, a lower platelet count at fever onset was associated with non-bleeding sepsis-related mortality and the need of organ support. Moreover, the trajectories of both neutrophil and platelet counts in the first 72 hours after the onset of febrile neutropenia were also associated with negative outcomes.

**Conclusion:**

These results highlight the heterogeneous role of platelets in the immune response and pave the way for studies exploring to what extent severe thrombocytopenia modulates the molecular and cellular mechanisms of post-febrile neutropenia sepsis in these patients.

## Introduction

Sepsis is a severe clinical condition that can be described as an unregulated systemic inflammatory response developed by the host in response to a pathogen [1]. In 2017 approximately 48.9 million incident cases of sepsis and 11 million sepsis-related deaths were recorded worldwide according to the Global Burden of Disease (GBD) study [2].

Sepsis manifests as an exaggerated inflammatory response in which tissue injury may be mediated by pathogens or by the host immune response. It involves several elements such as the complement system, coagulation cascade, endothelium, and circulating cells, including neutrophils and platelets [3]. Neutrophils and platelets seem to play a dual role contributing to both pathogen clearance and secondary tissue injury [4].

In patients with cancer, sepsis is frequently associated with chemotherapy-induced neutropenia and thrombocytopenia. Several studies have demonstrated that post-chemotherapy neutropenia is a risk factor for sepsis complications when compared to non-neutropenic sepsis [5,6]. However, the association of neutrophil counts with sepsis outcomes among patients with cancer and post-chemotherapy neutropenia is less evident [7,8]. Regarding platelet counts, to our knowledge, no studies have addressed the association of the magnitude of thrombocytopenia with the risk of febrile neutropenia (FN) in patients with hematological malignancies who typically present more severe thrombocytopenia than patients with other cancers. Moreover, most studies that explored the impact of neutrophil and platelet counts in FN considered only a single time-point analysis.

Neutrophils and platelets play critical roles in the host response to pathogens and act as putative mediators of secondary tissue injury in sepsis. Therefore, this study explored the association between the trajectories of these cellular counts within the first days of fever onset and clinical outcomes in a consecutive cohort of patients with hematological malignancies and post-chemotherapy neutropenia.

## Methods

### Study population

This was a retrospective study conducted at an academic tertiary hospital in Campinas, SP, Brazil. The study population included all patients with chemotherapy-induced neutropenia admitted between 2018 and 2021, identified through the laboratory database of complete blood counts (CBC). The filtering strategy involved: (i) identification of all results with an absolute neutrophil count (ANC) <1.0×10^9^/L; (ii) selection of the first CBC result that reached this level; (iii) selection of patients with a diagnosis of any hematological malignancies from the electronic medical records with a history of chemotherapy; and (iv) selection of patients with severe neutropenia (ANC <0.5×10^9^/L). Each individual medical record was then evaluated and patients without a history of fever during their admission were excluded. The study was approved by the local institutional review board, which waived the requirement for obtaining written informed consent from participants (#59359022.7.0000.5404), and it was performed according to the Declaration of Helsinki.

### Chemotherapy regimens and supportive treatment protocol

Patients were submitted to intensive chemotherapy regimens which included: induction or consolidation chemotherapy for acute leukemias, or salvage chemotherapy regimens for lymphoma and plasma cell neoplasms. Hematopoietic stem cell transplantation conditioning regimens were used as per protocol based on diagnosis. All admitted patients with FN received filgrastim until neutrophil recovery. An empirical antimicrobial regimen was initiated immediately after fever onset and modified upon pathogen identification or specific clinical criteria, in accordance with institutional and international protocols. Platelet and red blood cell transfusions were used as normally indicated in the context of post-chemotherapy cytopenias.

### Clinical and laboratory data, and study outcomes

Clinical and laboratory data were manually retrieved from the electronic medical records. FN was defined as the presence of fever (>37.8 °C) in patients with an ANC <0.5×10^9^/L. Sepsis was defined as the presence of life-threatening organ dysfunction in patients with clinical or laboratory evidence of infection [9]. The presence of these criteria was strictly adjudicated from patient records by one of the investigators (EVDP). The following outcomes were evaluated: sepsis-related death within 30 days from FN onset, septic shock, venous thromboembolism, need for mechanical ventilation, vasopressor agents or renal replacement therapy, and a composite outcome termed any organ support, defined as the need for any of the aforementioned supports. In order to facilitate interpretation, the neutrophil-to-platelet ratio (NPR) was calculated by multiplying the ratio of neutrophil to platelet counts (both in x 10^9^/L) by 100.

### Statistical analysis

Quantitative data were expressed as medians (with percentiles), minimum, maximum, mean, and standard deviation. Data distribution was assessed using appropriate statistical tests. Group comparisons were performed using t-tests or Mann-Whitney tests (2 groups), and ANOVA or Kruskal-Wallis tests (≥3 groups), depending on data normality. Correlations were evaluated using Pearson’s or Spearman’s tests. Diagnostic accuracy of neutrophil and platelet counts, and the neutrophil-to-platelet ratio were assessed by receiver operating characteristic (ROC) curve analysis and univariate/multivariate associations with primary and secondary outcomes. Statistical significance was set for p-values <0.05. Analyses were performed using Statistical Package for Social Sciences (SPSS) v. 25.0 and GraphPad Prism v8.0.

## Results

In total, 5,759 CBC results with neutropenia were identified during the study period, of which 113 were from patients with severe neutropenia, hematological malignancies and fever, as illustrated in the study flowchart (Supplementary Figure 1). Patient characteristics at the time of FN onset are shown in Table 1.

**Table 1:** Clinical and laboratory characteristics at the time of febrile neutropenia onset and outcomes (n = 113).

Characteristic	
Sex - n (male: female)	54:59
Age - median (IQR)	54.0 (37.5-60.0)
Diagnosis - n (%) Acute leukemia Lymphomas Plasma cell neoplasms Other	53 (46.9) 26 (23.0) 18 (15.9) 16 (14.2)
Comorbidity index - median (IQR)	2 (2-3)
Treatment modality - n (%) Intensive chemotherapy HSCT Other	78 (69.0) 24 (21.2) 4 (3.5)
qSOFA score - median (IQR)	0 (0-1)
ECOG performance status - median (IQR)	3 (3-3)
Neutrophil count (x 10^9^/L) - median (IQR)	0.04 (0.01-0.25)
Platelet count (x 10^9^/L) - median (IQR)	21 (10.0-55.5)
Neutrophil:Platelet ratio - median (IQR)	0.13 (0.03-0.84)
**Outcomes**	
Bloodstream infection (yes) - n (%)	38 (33.6)
Mechanical ventilation (yes) - n (%)	26 (23.0)
Renal replacement therapy (yes) - n (%)	10 (8.8)
Vasoactive drugs (yes) - n (%)	29 (25.7)
Septic shock (yes) - n (%)	25 (22.1)
Venous thromboembolism (yes) - n (%)	8 (7.0)
Any organ support (yes) - n (%)	38 (33.6)
30-day sepsis-related mortality - n (%)	19 (16.8)

HSCT: hematopoietic stem cell transplantation; qSOFA: quick sequential organ failure assessment; ECOG: (Eastern Cooperative Oncology Group Performance Status).

In the first 30 days following FN onset, sepsis-related mortality was 16.8% (n = 19). Of note, none of these nineteen patients died due to bleeding complications. The characteristics of patients stratified by two of the most relevant outcomes (sepsis-related mortality and need of any organ support) are shown in Supplementary Table 1.

**Figure 1:**
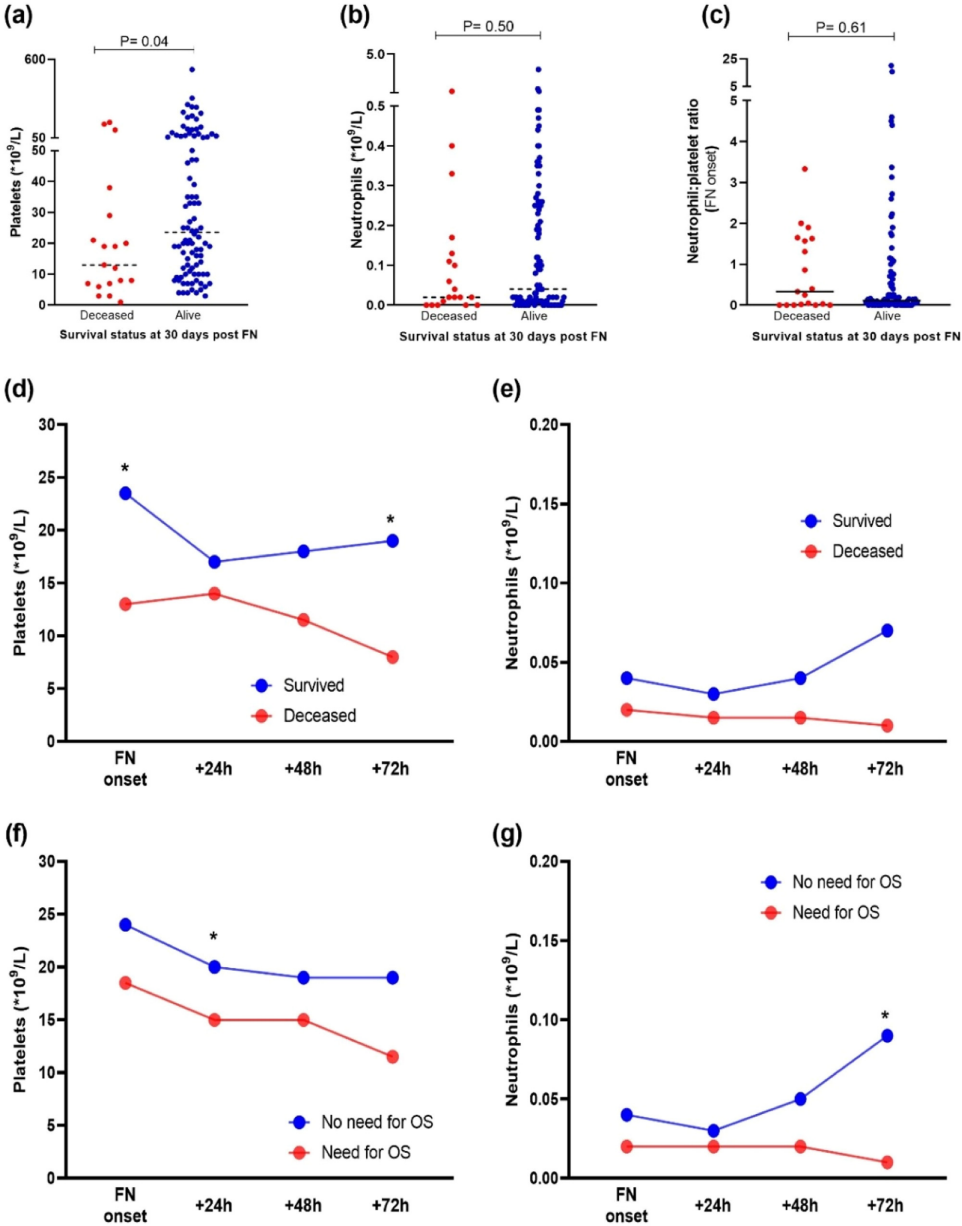
Dot blot and line graphs showing platelet count (a), neutrophil count (b), and neutrophil-to-platelet ratio (c) at the onset of febrile neutropenia (FN), stratified by 30-day survival status (Mann-Whitney test). Panels (d) and (e) depict the time course of the platelet and neutrophil counts, respectively, from FN onset up to 72 hours, according to survival status. Panels (f) and (g) show the time course of the platelet and neutrophil counts from FN onset up to 72 hours, according to the need for organ support (OS).

Neutrophil counts were similar between patients irrespective of sepsis-related mortality. In contrast, platelet counts were significantly lower in deceased patients (Figure 1 a-b), while no difference was observed in the NPR (Figure 1c).

Furthermore, the time-course of neutrophil and platelet counts were evaluated from the onset of FN up to 72 hours in patients stratified by relevant clinical outcomes. As shown in Figure 1d the trajectory of platelet counts was significantly different, with a downward trend in deceased patients and a more stable trajectory in patients who survived. A similar, albeit not significant, trend was observed in neutrophil counts (Figure 1e) when patients were stratified by survival status. In fact, when patients were stratified by the need of any organ support, the upward trend of neutrophil counts in the first 72 hours after FN onset in patients who survived was more evident and statistically significant.

**Figure 2:**
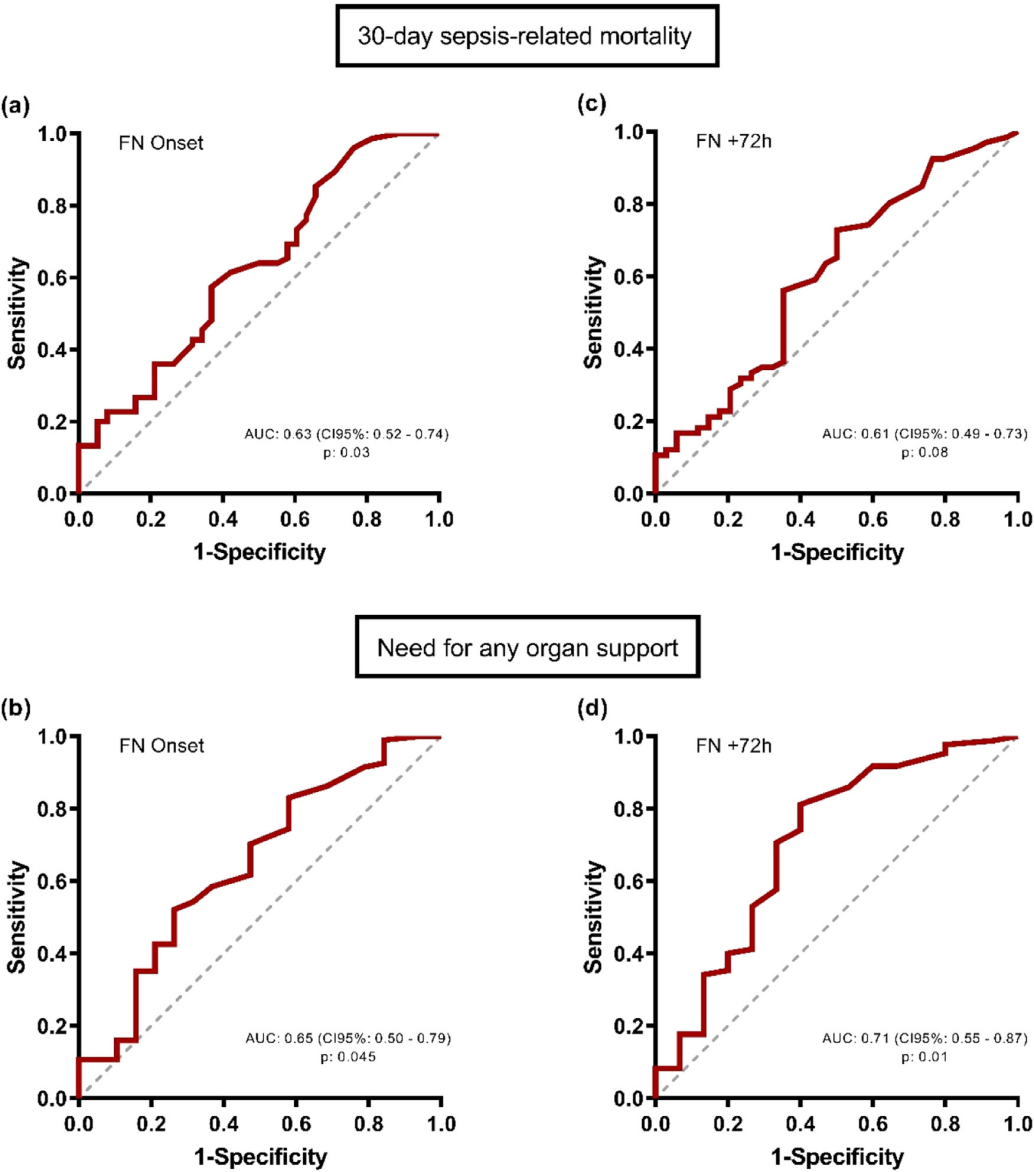
Diagnostic accuracy of platelet counts at fever onset and at 72 hours to predict clinical outcomes. Receiver operating characteristic curves of platelet count at fever onset (a-b) and after 72 hours (c-d) to predict 30-day sepsis-related mortality (Panels a and c) and need of any organ support (Panels b and d). FN: febrile neutropenia.

Since platelet, but not neutrophil counts, were significantly associated with 30-day sepsis-related mortality at the time of FN onset, the prognostic accuracy of platelet counts for the prediction of sepsis-related mortality and the need for any organ support in this population was explored. As shown in Figure 2, ROC curves demonstrate that the platelet count at the time of FN onset could significantly predict both outcomes (Figure 2a-b). Similar results were observed with the platelet count 72 hours after FN onset (data not shown). Using a cutoff of 13.0×10^9^/L identified by the ROC curve analysis, a significant difference is observed in the survival curve of the study population (Supplementary Figure 2).

**Figure 3:**
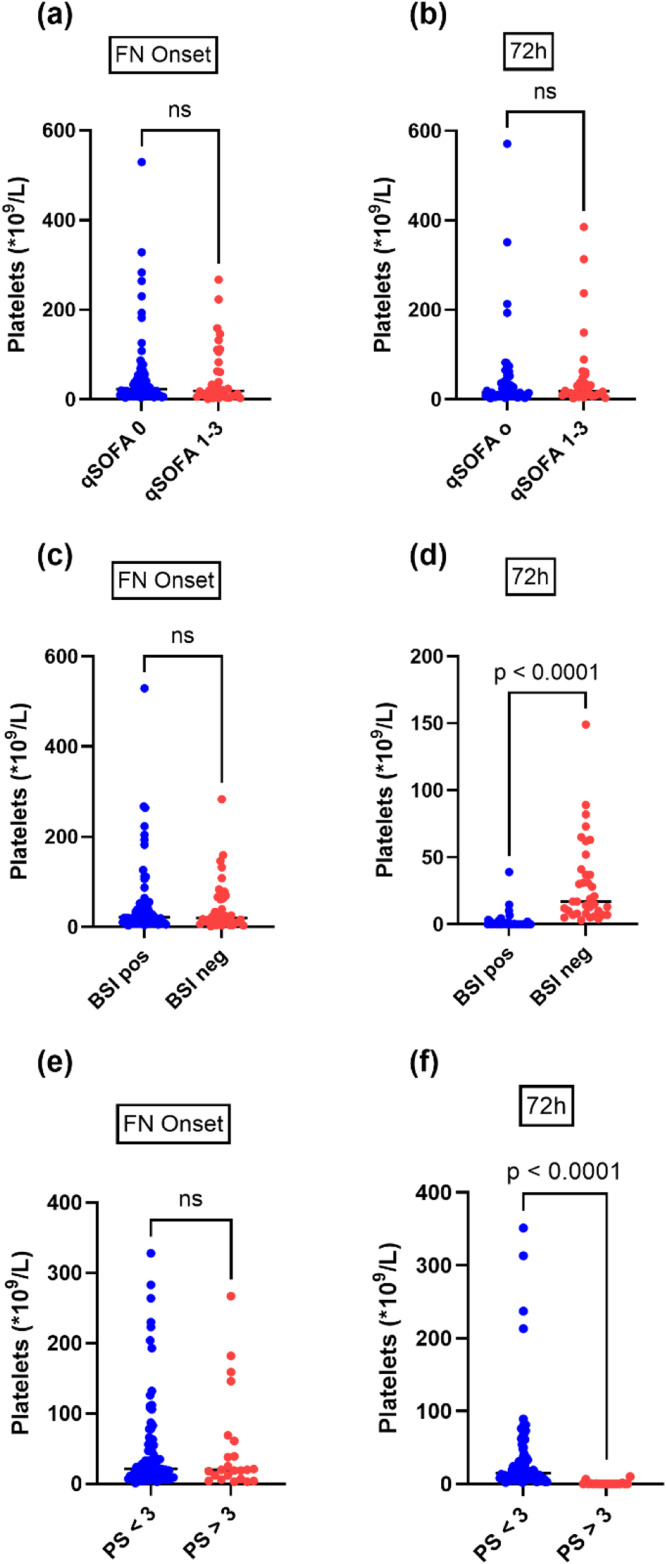
Dot blots of platelet counts at FN onset (left panels) and 72 hours after FN onset (right panels) stratified by quick sequential organ failure assessment (a-b), presence of bloodstream infection (BSI) (c-d) and performance status (PS) (e-f) (Mann–Whitney test). ns: non-significant.

Finally, to further investigate whether the association of platelet count at FN onset with mortality was dependent on disease severity, the association of platelet count with disease severity markers, namely quick sequential organ failure assessment (qSOFA), performance status and presence of bloodstream infections was analyzed. As shown in Figure 3, while this association was confirmed after 72 hours, suggesting that platelet counts are at least partly driven by disease severity, no such difference was present at fever onset (Figure 3).

## Discussion

Whether neutropenia influences outcomes in patients with hematological malignancies is still controversial. Moreover, the influence of thrombocytopenia, on sepsis-related outcomes among patients with hematological malignancies presenting chemotherapy-induced neutropenia has not been systematically explored. The main contribution of this study was to demonstrate that platelet counts at FN onset are associated with 30-day sepsis-related, non-bleeding mortality.

Despite the common association in clinical practice between neutropenia and sepsis severity, several studies concur with these results, corroborating the concept that neutropenia is not a reliable predictor of sepsis-related mortality in patients with hematological malignancies [7,8,10, 11, 12, 13, 14]. Accordingly, it has been emphasized that the sole presence of neutropenia should not be a criterion to prioritize the admission of patients to intensive care [15]. In addition, this study explores whether the time-course of neutropenia during the first 72 hours after fever onset provides additional prognostic information. This study demonstrated that while the neutrophil count at fever onset was not associated with sepsis severity, non-surviving patients exhibited a distinctly different kinetic trajectory of neutrophil recovery. Whereas surviving patients initiated neutrophil recovery within 48 hours of fever onset, deceased patients had persistently reduced counts, yielding a statistically significant difference in neutrophil levels by 72 hours post-FN onset. Whether a more protracted pattern of neutrophil recovery represents a consequence of a complicated clinical course that hampers the increment of neutrophils after FN onset, or a cause of the negative outcomes of these patients cannot be determined by our data, although the fact that the difference was observed only after 72 hours supports the former hypothesis.

In addition, the data of this study demonstrate an association between platelet counts and adverse sepsis-related outcomes. This relationship is well known, with the platelet count serving as one of the established biomarkers of organ failure in sepsis [16]. However, among patients who develop thrombocytopenia as a consequence of chemotherapy this association is less clear. In a comprehensive review that included 65 studies that explored predictors of outcomes in FN [17], only one study identified platelet count as a predictor variable; however, the study included all types of cancer, with thrombocytopenia (platelet count <50×10^9^/L) being considered a categorical variable [18]. Two previous retrospective cohort studies also reported an association between thrombocytopenia and worse outcomes after FN but both included all types of cancer patients and considered thrombocytopenia (platelet count <50×10^9^/L) as a categorical variable [19,20]. Hematological malignancies, which require chemotherapeutic regimens that more frequently induce severe thrombocytopenia, are a well-known predictor of FN severity when associated with fungal infections [21]; consequently, it cannot be excluded that the association observed in these studies could be driven by cancer type. Notably, none of the 19 FN-related deaths included in the present analysis were attributed to bleeding, suggesting that thrombocytopenia could negatively impact other mechanisms of the innate immune response. This raises the possibility that severe thrombocytopenia could affect platelet-mediated vascular protection in inflammatory settings, as shown in *in vivo* studies [22,23]. However, a probable hypothesis is that thrombocytopenia results, at least in part, from a more complicated clinical course in these patients. Regarding the trajectory of platelet counts, a similar pattern to the one observed for neutrophil counts was noted, with patients who eventually died from sepsis complications presenting a more protracted recovery of platelet counts. Again, whether this represents a cause or consequence of their clinical course remains to be determined.

Another goal of this study was to explore whether the NPR could provide more precise prognostic information, based on the importance of the interaction between these cells in the physiopathology of host response to sepsis. This hypothesis was not confirmed.

Limitations of this study include those inherent to retrospective designs, such as missing data and recall bias. To minimize these, medical records were reviewed using a standardized form on the Research Electronic Data Capture (RedCap) platform. Additionally, the primary outcomes (mortality and need for organ support) are less prone to such biases. The relatively small sample size limited the feasibility of classical multivariate analysis. To address this, a stratified analysis of platelet counts by severity (qSOFA: 0 vs. 1–3; ECOG: <3 vs. ≥3, and BSI presence vs. absence) was performed. A statistically significant difference in platelet counts was observed at 72 hours, which likely reflects the progression of infection and sepsis, leading to thrombocytopenia as described in the literature. In contrast, at FN onset no significant differences in platelet counts were observed across any of the severity stratifications. This suggests that the platelet count at FN onset may display some degree of independence from these severity indicators. Although this independence may not be absolute, the absence of differences at FN onset followed by their emergence at 72 hours supports the interpretation that thrombocytopenia becomes progressively influenced by the worsening infectious and septic process over time. Nonetheless, one strength of this study was the analysis of a consecutive cohort of neutropenic and severely thrombocytopenic patients with hematological malignancies and FN, whose baseline and clinical characteristics aligned with the literature. Uniquely, this study evaluated the trajectory of neutrophil and platelet counts, enhancing the robustness of the findings compared to previous studies based solely on a single value at fever onset.

Another limitation is that the precise timeframe between chemotherapy and FN was not recorded, although based on the expected kinetics of neutrophil depletion and recovery after the chemotherapy regimens considered in the study, the nadir of neutrophil count occurs 7-14 days after chemotherapy. Moreover, platelet transfusions were not included in the analysis, which may be a confounding factor. However, since transfusions followed the contemporary standard protocols, we believe that platelet counts used in the analyses illustrate the dynamics of platelet count variations in patients submitted to intensive chemotherapy. Furthermore, the precise time between fever onset and antimicrobial administration was not recorded, which is a well-characterized prognostic factor in FN.

In conclusion, a low platelet count at the time of fever onset was associated with sepsis-related mortality and other negative outcomes in a population of patients with FN and hematological malignancies, independent of bleeding. Neutrophil counts at the time of FN were not associated with sepsis-related mortality or other negative outcomes, although trajectories of both platelet and neutrophil counts within the first 72 hours after FN onset were associated with sepsis mortality. These results highlight the heterogeneous role of platelets in the immune response [24], and pave the way for studies exploring to what extent severe thrombocytopenia modulates the molecular and cellular mechanisms of post-NF sepsis in these patients.

## Funding sources

This study was funded by the Sao Paulo Research Foundation (FAPESP), grants 2023/04541-8, 2022/13216-0, 2021/12945-6, 2023/03765; and Capes Finance code 01.

## Data availability

The data that support the findings of this study are available from the corresponding author upon reasonable request.

## Conflicts of interest

The authors declare no competing interests.

## Appendix Supplementary materials

Image, application 1
