# Supplementary material for: Platelet counts, but not neutrophil counts, are associated with non-bleeding-related mortality in patients with hematological malignancies and post-chemotherapy febrile neutropenia
Source: Hematol Transfus Cell Ther. 2026 Jul 10;48(3):106498. doi: 10.1016/j.htct.2026.106498 (PMC13381977; doi:10.1016/j.htct.2026.106498)
Supplement: Supplementary file 1 [file mmc1.docx]

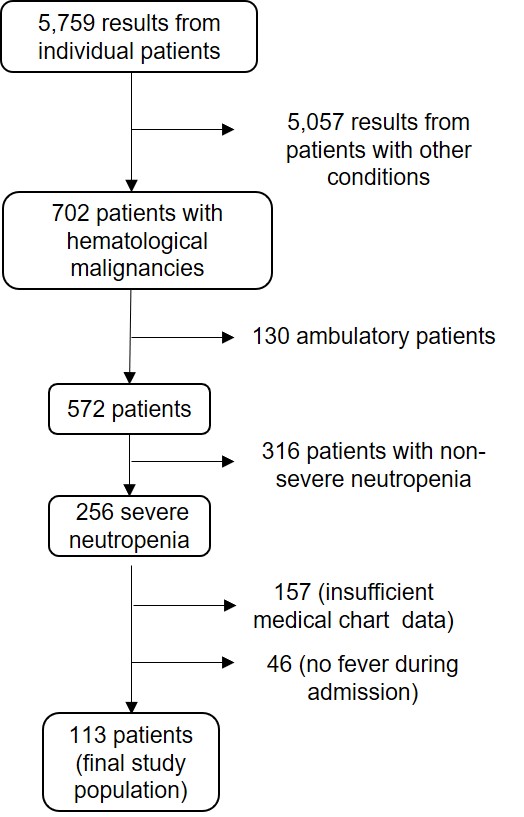


**Supplementary Figure 1:** Study flowchart demonstrating the selection strategy of the study population. Medical chart data were considered insufficient to include patients when complete blood count results were not accompanied by any clinical and outcome data

**Supplementary Table 1:** Comparison of the study population according to clinical outcomes

| **Sepsis-related mortality**  30-day survival status | **Deceased** | **Alive** | **P-value** |
| --- | --- | --- | --- |
| **Sex** (male: female) - n | 9:10 | 45:49 | NS |
| **Age (years) –** median (IQR) | 48.0 (30.0-55.0) | 55.5 (41.0-61.0) | NS |
| **Diagnosis** - n (%)  Acute leukemia  Lymphomas  Plasma cell neoplasms  Other | 10 (52.6)  5 (26.3)  0 (0.0)  4 (21.1) | 43 (45.7)  21 (22.3)  18 (19.1)  12 (12.8) | NS |
| **Comorbidity index –** median (IQR) | 2 (2-3) | 2 (2-3) | NS |
| **Treatment modality** - n (%)  Intensive chemotherapy  HSCT  Other | 14 (73.7)  4 (21.1)  1 (5.3) | 64 (69.8)  20 (21.3)  3 (3.2) | NS |
| **qSOFA score** – median (IQR) | 1 (0-1) | 0 (0-1) | NS |
| **Neutrophils (**x 10^9^/L) – median (IQR) | 0.02 (0.00-0.13) | 0.04 (0.01-0.25) | 0.501 |
| **Platelets (**x 10^9^/L) – median (IQR) | 13.0 (7.0-29.0) | 23.5 (10.75-61.5) | **0.045** |
| **Neutrophil:platelet ratio** – median (IQR) | 2.5 (0.31-14.5) | 0.12 (0.03-0.72) | 0.600 |
| **Bloodstream Infection (**yes:no) - n | 10:8 | 28:65 | **0.037** |
| **ECOG PS** – median (IQR) | 4 (3-4) | 3 (3-3) | **<0.001** |
| **Need of any organ support*** | **Yes** | **No** | **P-value** |
| **Sex** (male: female) - n | 15:23 | 39:36 | NS |
| **Age** – median (IQR) | 54.5 (43.25-61.25) | 53.0 (37.0-60.0) | NS |
| **Diagnosis** - n (%)  Acute leukemia  Lymphomas  Plasma cell neoplasms  Other | 20 (52.6)  9 (23.7)  4 (10.5)  5 (13.2) | 33 (44.0)  17 (22.7)  14 (18.7)  11 (14.7) | NS |
| **Charlson comorbidity index** – median (IQR) | 3 (2-4) | 2 (2-3) | 0.054 |
| **Treatment modality** - n (%)  Intensive chemotherapy  HSCT  Other | 28 (73.7)  7 (18.4)  1 (2.6) | 50 (66.7)  17 (22.7)  3 (4.0) | NS |
| **qSOFA score** – median (IQR) | 1 (0-1) | 0 (0-0.25) | **0.013** |
| **ECOG PS** – median (IQR) | 3 (3-4) | 3 (3-3) | **<0.001** |
| **Neutrophils (**x 10^9^/L) – median (IQR) | 0.02 (0.0-0.23) | 0.04 (0.01-0.25) | 0.491 |
| **Platelets (**x 10^9^/L) – median (IQR) | 18.5 (6.75-38.25) | 24 (12.0-64.0) | **0.028** |
| **Neutrophil:Platelet ratio** – median (IQR) | 0.25 (0.0-1.44) | 0.10 (0.04-0.76) | 0.669 |
| **Bloodstream Infection (**yes:no) - n | 20:17 | 18:56 | 0.002 |

HSCT: hematopoietic stem cell transplantation; qSOFA: quick sequential organ failure assessment; ECOG PS: ECOG performance status; NS: Non-significant

Any organ support was defined as the need for mechanical ventilation or renal replacement therapy or vasopressor agents.


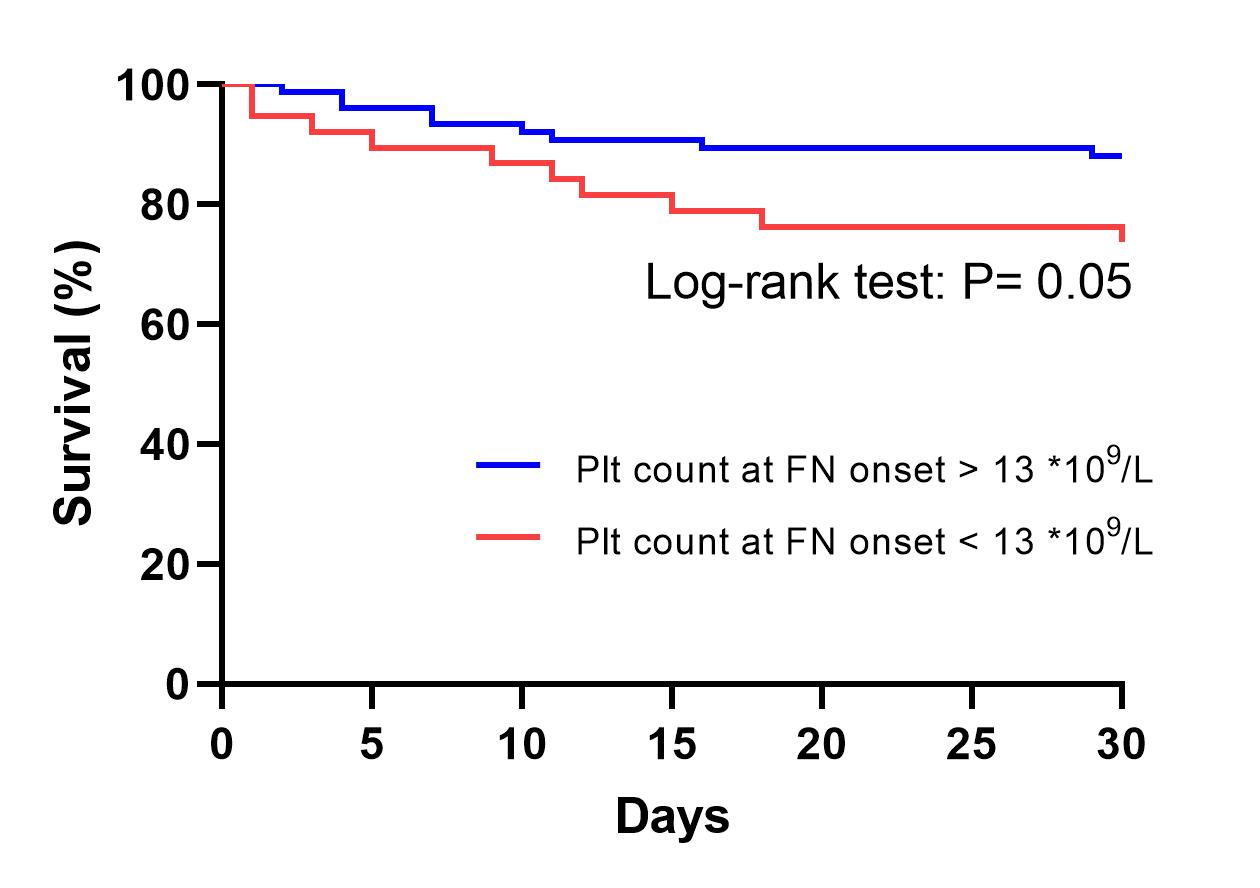


**Supplementary Figure 2:** Kaplan Meyer curves comparing 30-day sepsis-related mortality in patients stratified by platelet count at the time of febrile neutropenia onset (Log-rank test).
